# Cardiac arrest centers – the future?

**DOI:** 10.1186/1471-227X-15-S1-A18

**Published:** 2015-06-24

**Authors:** Christian Storm

**Affiliations:** 1 Department for Internal Medicine, Nephrology and Intensive Care, Cardiac Arrest Center of Excellence, Charité-Universitätsmedizin Berlin, Germany

## 

Target temperature management should be state of the art nowadays after cardiac arrest. With gaining knowledge about reperfusion injury after general hypoxia it became obvious that intensive care treatment contains a bundle of different steps rather than only cooling the patient. In trauma patients, outcome was improved by treatment in high-volume centers with high experience [1,2]. Concerning the overall outcome and length of stay, trauma patients benefit from admission to a trauma center. However, current data show a higher survival rate after cardiac arrest if hospitals treat a large number of survivors [3]. In addition, hospital characteristics such as the capability for a coronary intervention (PCI) 24/7 were shown to have a significant impact on outcome after cardiac arrest [4]. Therefore, the foundation of cardiac arrest centers (CAC) in Germany, similar to the USA, is the corollary [5]. The mission of CAC is manifold. Owing to the high number of patients treated, a written standard protocol and a data registry are essential. High quality and quality management need to be transparent and reviewable at any time. Besides a structured admission process including, for example, the timing of PCI and/or CT scan, the neurological prognostication process should also follow a predefined, written timeline and standardized diagnostic steps, as this is a very sensitive and complex part of the treatment. Furthermore, CAC are preset for research and participation in international multicenter trials. Especially in cardiac arrest patients, future questions will only be answered with a high number of patients due to heterogeneity in cause of arrest and grade of hypoxia. Potential additional costs have to be part of a novel reimbursement structure concerning post-cardiac arrest therapy.

CAC are therefore necessary and needed in the near future.

## Financial disclosure

CS has received honorarium and/or travel costs from Medivance, Zoll, C. R. BARD, Philips, EMCOOL, COVIDIEN and Nonin; and financial or material support for research from Medivance, Zoll, C. R. BARD, EMCOOL, COVIDIEN, Nonin and Deutsch Stiftung für Herzforschung.

## Declaration

This abstract and supplement was proposed, developed and commissioned by BMC Emergency Medicine and was funded by an educational grant from C. R. BARD, NJ, USA. The published abstract was independently prepared by the author. C. R. BARD had no input into the content.

## References

[B1] Nathens AB Jurkovich GJ Maier RV Grossman DC MacKenzie EJ Moore M Relationship between trauma center volume and outcomes JAMA 2001 285 1164 71 10.1001/jama.285.9.1164 11231745

[B2] MacKenzie EJ Rivara FP Jurkovich GJ Nathens AB Frey KP Egleston BL A national evaluation of the effect of trauma-center care on mortality N Engl J Med 2006 354 366 78 10.1056/NEJMsa052049 16436768

[B3] Callaway CW Schmicker R Kampmeyer M Powell J Rea TD Daya MR Receiving hospital characteristics associated with survival after out-of-hospital cardiac arrest Resuscitation 2010 81 524 9 10.1016/j.resuscitation.2009.12.006 20071070 PMC2856722

[B4] Wnent J Seewald S Heringlake M Lemke H Brauer K Lefering R Choice of hospital after out-of-hospital cardiac arrest – a decision with far-reaching consequences: a study in a large German city Crit Care 2012 16 R164 10.1186/cc11516 22971320 PMC3682259

[B5] Kill C, FN Scholz J Scholz KH Andresen D Busch HJ Die spezialisierte Krankenhausbehandlung nach erfolgreicher Wiederbelebung ist überlebenswichtig Notfall Rettungsmedizin 2014 17 331 2 10.1007/s10049-014-1889-9

